# Implantable Optrode Array for Optogenetic Modulation and Electrical Neural Recording

**DOI:** 10.3390/mi12060725

**Published:** 2021-06-19

**Authors:** Saeyeong Jeon, Youjin Lee, Daeho Ryu, Yoon Kyung Cho, Yena Lee, Sang Beom Jun, Chang-Hyeon Ji

**Affiliations:** 1 Department of Electrical and Computer Engineering, Seoul National University, Seoul 08826, Korea; saeyoung2425@naver.com (S.J.); daehor@gmail.com (D.R.); 2 Department of Electronic and Electrical Engineering, Ewha Womans University, Seoul 03760, Korea; iamugin@gmail.com (Y.L.); choyoon90@gmail.com (Y.K.C.); yena0121@gmail.com (Y.L.); juns@ewha.ac.kr (S.B.J.); 3 Graduate Program in Smart Factory, Ewha Womans University, Seoul 03760, Korea; 4 Department of Brain and Cognitive Sciences, Ewha Womans University, Seoul 03760, Korea

**Keywords:** optogenetics, optrode, electrophysiology, electrode, channelrhodopsin

## Abstract

During the last decade, optogenetics has become an essential tool for neuroscience research due to its unrivaled feature of cell-type-specific neuromodulation. There have been several technological advances in light delivery devices. Among them, the combination of optogenetics and electrophysiology provides an opportunity for facilitating optogenetic approaches. In this study, a novel design of an optrode array was proposed for realizing optical modulation and electrophysiological recording. A 4 × 4 optrode array and five-channel recording electrodes were assembled as a disposable part, while a reusable part comprised an LED (light-emitting diode) source and a power line. After the characterization of the intensity of the light delivered at the fiber tips, in vivo animal experiment was performed with transgenic mice expressing channelrhodopsin, showing the effectiveness of optical activation and neural recording.

## 1. Introduction

For more than a decade, optogenetics has been widely employed to investigate a variety of neural mechanisms in neuroscience studies [1,2,3,4,5,6,7,8,9]. Unlike conventional electrophysiological methods, optogenetic techniques utilize light to control the activity of genetically modified neurons expressing light-activated opsin proteins. Therefore, it enables the precise modulation of genetically selected neurons in complicated neural circuits [6,10]. As the optogenetic technique became popular, the need for simultaneous neural recording has also grown in order to confirm optical neuromodulation. An integrated device that can simultaneously deliver light stimuli to activate or inhibit specific neuronal cells and record the neural response activities has been highly demanded [9,11,12,13,14,15,16,17,18]. As this micro-electro-mechanical systems (MEMS) technology advances, optical stimulation and recording system configuration can be fabricated on a microscale [19,20,21,22,23,24,25]. Therefore, a MEMS-based optrode is considered to be one of the promising optogenetic platforms.

As an optogenetic light source, light-emitting diodes (LEDs) and lasers are commonly used in most applications [14,22,26,27,28,29]. High intensity and a narrow focus of light are required for optogenetic experiments. Compared to LED, lasers can provide very high intensity in a focused spot and have a narrow spectral width. However, there is a critical issue that the system cannot be miniaturized, and tissue damage can occur due to the high output power of the laser [30,31]. On the other hand, the light intensity of an LED can be easily modulated without concern for stability or tissue damage [28]. An LED is also suitable for freely behaving optogenetics experiments because it has a long lifetime, low power consumption, and low cost. For these reasons, in this study, an LED was chosen as the light source.

The utility of the optical system was verified in previous studies in terms of light intensity, heat dissipation, and spatial light distribution [21,25]. In this study, a new optrode array integrated with electrophysiological recording electrodes was proposed so that the neural activity from adjacent neurons evoked by optogenetic stimulation could be recorded. The proposed system comprises disposable and reusable parts. The disposable bottom part includes multi-channel recording electrodes and square-shaped glass microlenses with an optical fiber array. The reusable upper part consists of an LED light source and external power supply lines. After in vivo experiments, the reusable part can be easily detached and applied to another animal, while the disposable part is permanently implanted. The disposable part is protected by a plastic cover that can be firmly attached using magnetic attraction. To prove the feasibility of the proposed system, the optrode array was implanted into the brains of transgenic mice expressing channelrhodopsin-2 (ChR2) in Ca^2+^/calmodulin-dependent protein kinase II-positive neurons (CaMKIIα::ChR2 mouse). The neural activity from individual neurons was successfully recorded using the recording electrodes while optically stimulating the neurons. The whole system can be implemented further to a wireless system, which stimulates and records brain signals by adopting a low-power RF system on a chip. The wireless part controls the LED light pulse parameters and gathers signals through instrumentation amplifiers.

## 2. Fabrication

### 2.1. Microlens Array and Through-Silicon via Fabrication

**Figure 1:**
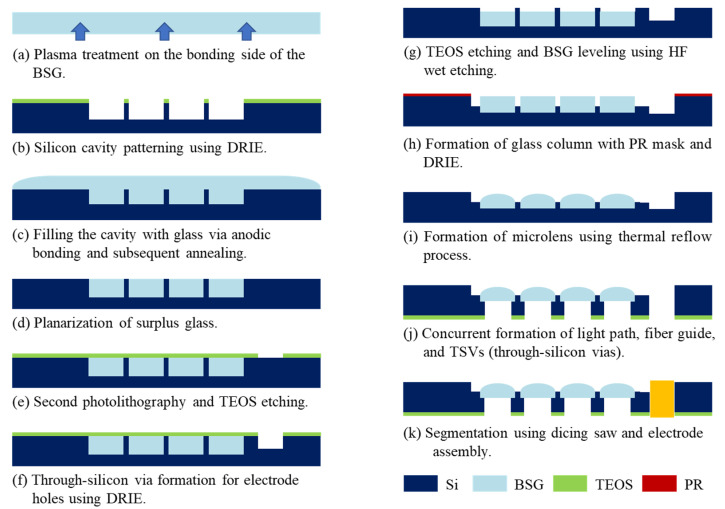
The fabrication process of the microlens array (MLA) and through-silicon vias (TSVs).

Figure 1 shows the overall fabrication process of the microlens array (MLA) with through-silicon vias (TSVs). The device was fabricated with bulk micromachining technology. The process began with the plasma pre-treatment on the bonding side of the borosilicate glass (BSG, Borofloat 33, Schott AG, Mainz, Germany) substrate, which was attached to the silicon substrate. The pre-treatment process was performed with a reactive ion etcher with 50 sccm of chlorine gas and radio frequency (RF) power of 150 W for 3 min (Figure 1a). The plasma treatment is essential for suppressing surface crystallization, which can affect the optical properties of the microlens [32]. After the 490 μm deep cavity formation on the 1 mm thick double-side-polished silicon wafer using the TEOS (tetraethoxysilane) silicon oxide etch mask and deep reactive ion etching (DRIE), the cavities were filled with molten BSG using anodic bonding and a subsequent annealing process at 850 °C for 8 h (Figure 1b,c). The surface of the silicon substrate was planarized by removing the surplus BSG remaining on top of the cavities using the CMP (chemical mechanical polishing) process (Figure 1d). As shown in Figure 1e,f, the second photolithography, oxide etching, and DRIE processes were carried out to form the half-etched holes for TSV formation. After the oxide mask layer was removed using a dry oxide etching process, the wafer was immersed in a 49% hydrofluoric acid solution for 6 min and 10 s to reduce the height of the glass columns from 490 μm to 453 μm (Figure 1g). The second DRIE process was performed with a photoresist mask to form the square-shaped glass columns partially protruding above the silicon surface. A negative thick photoresist mask (DNR-L300, Dongjin, Jincheon-gun, Korea) was spin-coated at 2000 rpm for 40 s, and soft baking and post-exposure baking were performed at 100 °C for 90 s (Figure 1h). The height of the glass columns protruding above the silicon surface was 35 μm. After the second thermal reflow process at 850 °C for 40 min, the square-shaped glass MLA array was formed on the silicon substrate (Figure 1i). Figure 1j shows the backside DRIE process with an oxide mask that formed the vias for the optical fibers aligned with the center of the glass MLA and the TSVs for the recoding electrode insertion. Finally, the silicon die was diced into 5 × 5 mm^2^ dies and assembled with the recording electrodes (Figure 1k).

### 2.2. MEMS Optrode Array Assembly

**Figure 2:**
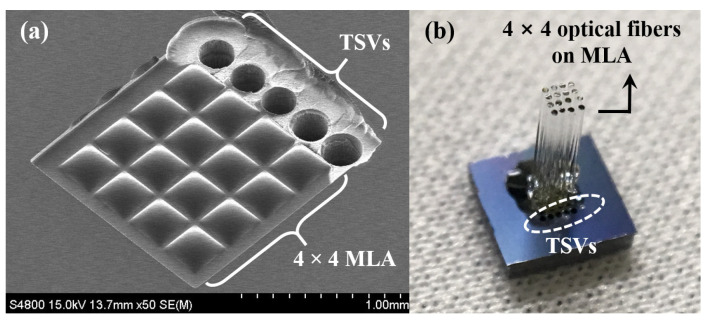
Fabrication results: (**a**) fabricated MLA and TSV; (**b**) 4 × 4 optical fiber array assembled with the MLA.

The scanning electron microscope (SEM) image of the fabricated 4 × 4 MLA and TSVs on a 5 × 5 mm^2^ silicon die is shown in Figure 2a. The designed dimension of each microlens was 300 × 300 µm^2^ with a 340 µm center-to-center distance. The TSV had a 250 µm diameter and 300 µm center-to-center distance. The optical fibers were manually assembled by inserting the fibers into the vias formed under the MLA with a guide jig that was specifically designed for the alignment and fixation of the optical fiber array (Figure 2b). The lengths of the optical fibers and the recording electrodes protruding from the bottom of the disposable part were 4 mm and 5.2 mm, respectively.

**Figure 3:**
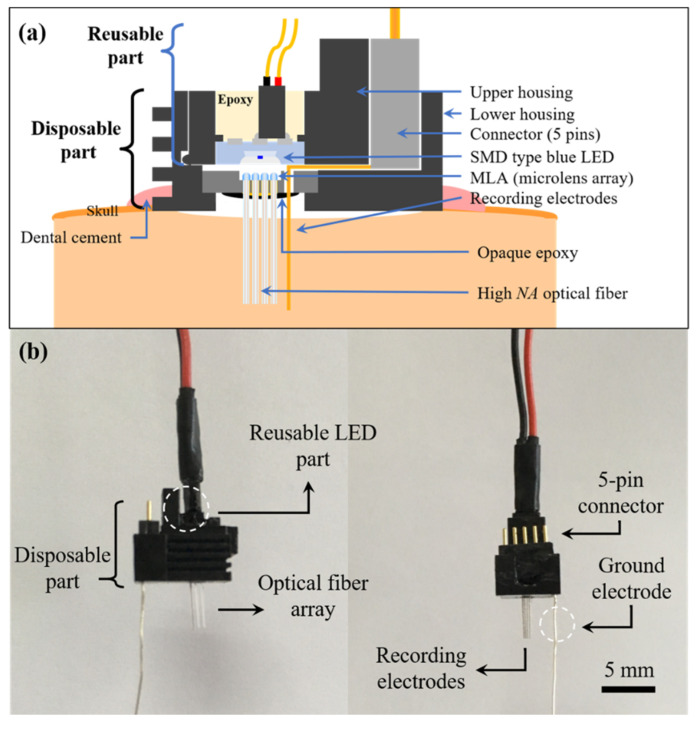
(**a**) Conceptual diagram of the proposed system for optical stimulation and electrophysiological recording; (**b**) fully assembled micro-electro-mechanical systems optrode array (MOA).

Figure 3a depicts the proposed MEMS optrode array (MOA) design in a cross-sectional view. The device can be separated into two components, which are the reusable part and the disposable part. The reusable part, which is the light source for the system, consists of an SMD (surface mounted device) type blue LED (LB G6SP, OSRAM, Sunnyvale, CA, USA) with a 469 nm wavelength, the upper housing, and the external power supply lines. The LED is located at the end of the upper housing, and the center of the LED is aligned with that of the MLA for high light delivery efficiency. The wavelength of light incident on the MLA can be changed simply by replacing the LED with one that has a different wavelength to match the wavelength required by specific target opsins. The disposable part comprises a 4 × 4 glass MLA and a 4 × 4 array of high NA optical fibers (FP200URT, Thorlabs, Inc., Newton, NJ, USA) that are passively aligned with the MLA and the recording electrodes. In the previous study, a 3.14 dB enhancement of output light intensity was confirmed when using the MLA and optical fiber array configuration used in this study [25]. Passively assembled optical fibers were fixed to the vias located at the bottom of each microlens using an index-matching epoxy (353ND, Epoxy Technology, Billerica, MA, USA). After the assembly, an opaque epoxy (832B, MG Chemicals, Burlington, ON, Canada) was applied to the bottom side of the silicon substrate to block the leakage light from the gap between the vias and optical fibers. The PFA (perfluoroalkoxy)-coated tungsten wires (A-M System, Sequim, WA, USA) are used for recording electrical signals, and the wires were directly connected to a 5-pin connector. The diameter of the electrode was 101.6 µm, and the impedance was less than 1 MΩ at 1 kHz. Five TSVs were formed near the 4 × 4 MLA, and the recording wires were inserted into each TSV for passive alignment with the optical fiber and fixation. At the front side of the customized polycarbonate upper and lower housings, two circular magnets with 2 mm diameter and 1 mm thickness were used with the latch structure for mechanical snap-in assembly. Figure 3b shows the fully assembled MOA device. The total dimension of the MOA device was 7 × 10 × 5 mm^3^.

The MOA is directly inserted into the target area of the brain, and the disposable part is fixed to the skull using dental cement. When the LED is turned on, the light passes through the MLA and the optical fiber array and reaches the fiber tip to illuminate the target region. In the previous study, it was shown that the temperature increase at the optical fiber tip was less than 0.5 °C, and the relative light intensity remained above 91% up to a distance of 2 mm from the tip [21]. The excitation parameters can be controlled by changing the frequency, pulse width, application time, and input current using the external power supply.

## 3. Experimental Results

### 3.1. Optical Characterization

**Figure 4:**
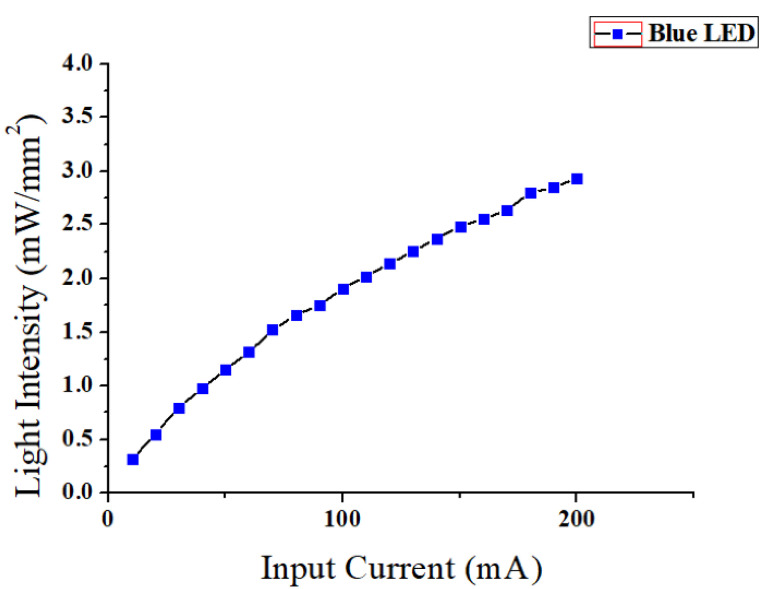
Measurement result of the light intensity of the fabricated device.

Figure 4 shows the light intensity measurement result of the fabricated device. The measurement setup consisted of an optical power meter (PM100D, Thorlabs Inc., Newton, NJ, USA), a photodiode power sensor (S121C, Thorlabs Inc., Newton, NJ, USA), and a four degree-of-freedom (DOF) manual microstage. The measurement was conducted in the presence of ambient light. The position of the device was adjusted using the microstage to align the tip of the optical fibers to the measurement part of the photodiode sensor. A DC input current was applied to the device. Although not perfectly linear, the measured light intensity of the device increased as the input current increased. The maximum measured light intensity was 2.936 mW/mm^2^ at a dominant wavelength of 469 nm when the applied current was 200 mA. The minimum light intensity of 1 mW/mm^2^ required to excite the target photosensitive molecule, namely, ChR2 (channelrhodopsin-2), could be covered with input current ranging from 50 to 200 mA [4]. The measured light delivery efficiency of the device was −7.7 dB.

### 3.2. In Vivo Animal Experiment Protocol

**Figure 5:**
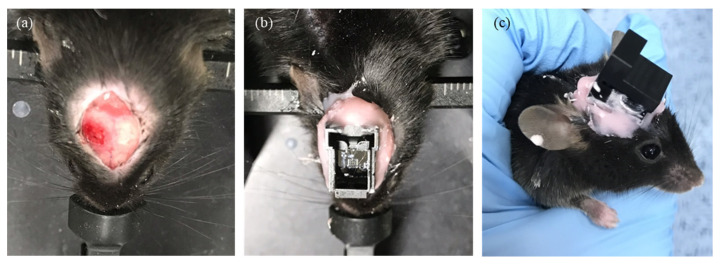
MOA implantation surgery: (**a**) target brain area was exposed by drilling into the skull; (**b**) the MOA was inserted and secured to the skull with dental cement; (**c**) the disposable part of the MOA was covered with a plastic cover during recovery.

In order to test the efficiency of the MOA, hGFAP-ChR2 mice, which are an astrocyte-specific gene-targeting transgenic model, was used (Figure 5a). Tamoxifen (100 μL/20 g) was administered to each transgenic mouse via an intraperitoneal (i.p.) injection per day. Two weeks after the tamoxifen induction, the mouse expressed ChR2 in astrocytes. The animal care and surgical procedures were approved by the Institutional Animal Care and Use Committee (IACUC) at the Ewha Womans University (no. 20-029). Mice were anesthetized with a ketamine–xylazine cocktail at an initial dose of 0.1 mL/g using an i.p. injection. The MOA was inserted into the hippocampus (AP: −1.8 to −2.8 mm, ML: 0.5–2.5 mm, DV: −1 to −2 mm) and fixed with dental cement (Figure 5b). For the long-term in vivo study, only the disposable part of MOA remained with the plastic cover when the system was not used for consecutive days to protect the MLA (Figure 5c).

Optical stimulation with blue (469 nm) LED light was delivered to activate the neural pathway. Arduino was used to drive the LED light source, and the output power of the LED was controlled using a function generator and power supply. The light pulse had a 4% duty cycle with a 4 ms pulse duration, and the frequency was 10 Hz. Optical stimulation was applied for two seconds during neural recording. In order to confirm the effect of light-induced neural activities, the individual neural spike activities were detected, sorted, and counted before, during, and after stimulation for two seconds each. If LED blue light stimulated neurons in the hippocampus region, the connected neural pathway would be activated, and the evoked neural activities of neighboring neurons could be recorded using multi-channel recording electrodes.

Raw neural signals (0.1–20 kHz bandpass filtered, 60 Hz notch filtered) were recorded at a 20 kHz sampling rate and amplified and digitized using an Intan RHD2132 headstage. The recorded data were analyzed with MATLAB (version 9.4.0, R2018a, MathWorks Inc., Natick, MA, USA).

### 3.3. Optogenetic and Electrophysiological Experiments

**Figure 6:**
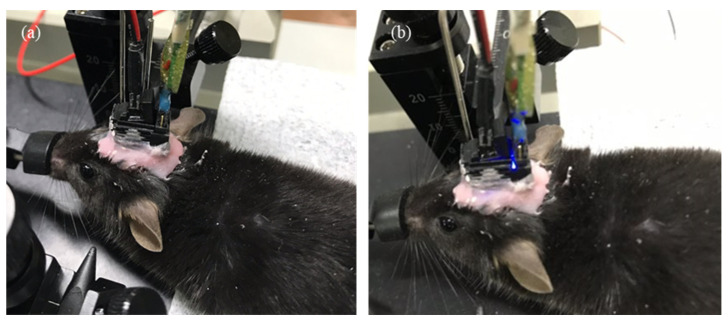
The result of optical stimulation: (**a**) LED light off and (**b**) LED light on.

**Figure 7:**
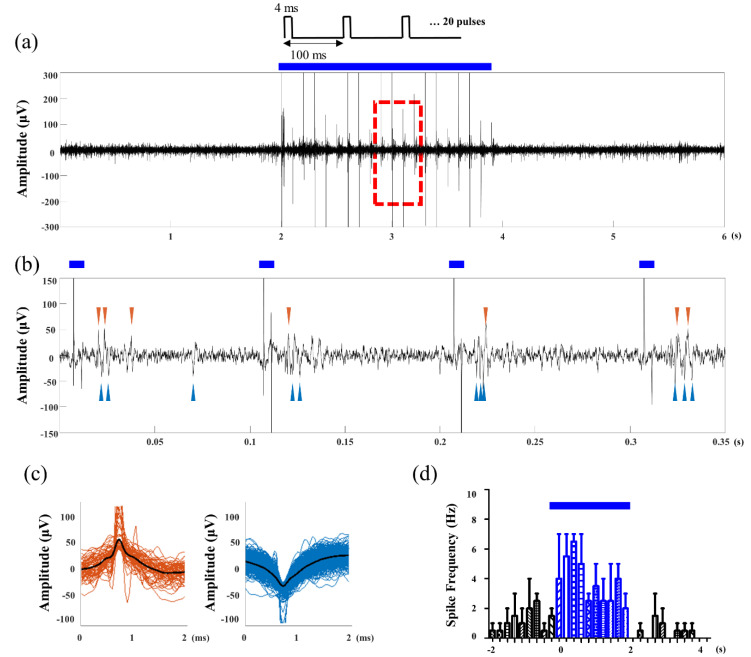
Electrophysiological recording results: (**a**) raw data of the neural recording; (**b**) zoomed waveform showing stimulation artifacts and evoked spikes; (**c**) the sorted spike waveform of two different neurons; (**d**) spike histogram. Throughout the figures, blue bars indicate the time window of the optical stimulation.

After the MOA implant surgery, optical stimulation and neural recording were conducted to verify that the MOA is suitable for in vivo optogenetic neuromodulation. Optical stimulation was controlled using Arduino. A 125 mA alternating current was applied for the stimulation, and the light intensity was 2 mW/mm^2^. LED blinking and the light artifact was observed according to programmed pulse parameter (Figure 6 and Figure 7a). The electrophysiological recording was performed simultaneously with light stimulation on neurons. The fabricated MOA successfully recorded the neural signals. Spontaneous and light-induced activities of hippocampal neurons were detected by multi-channel recording electrodes in the MOA (Figure 7a). Neural activity increased during optical stimulation compared to the spontaneous activity before the stimulation (Figure 7b). Two different spike waveforms were extracted from the recording signal via spike sorting, and both spike waveforms were induced after optical pulses (Figure 7c,d). Two different spike waveforms were extracted from the recording signal (Figure 7). A long-term in vivo test was possible because the plastic cover protected the MLA. It was demonstrated that the signal was stably measured two weeks after the implant surgery.

## 4. Conclusions and Discussion

In this study, a new design of an optrode array for optogenetic neuromodulation and neural recording was proposed. The device was fabricated using MEMS technologies, including TSVs for the aligned assembly of optical fibers and recording electrodes. The system was implanted into the brain of transgenic mice and delivered light stimulation to the target brain region. Based on the electrophysiological recording experiment, the neural activities were successfully detected via the assembled metal electrode array from individual neurons. Despite the successful result of the in vivo experiment, the close juxtaposition of optical fibers might cause tissue damage in the brain area of interest. However, the proposed fabrication method provides high design flexibility, including in terms of the length, interspacing, and number of optical fibers or electrodes; fiber tip shape; and the relative position between optical fibers and electrodes. Therefore, in further study, the design parameters can be adjusted (i.e., increasing spacing) to minimize the tissue damage and will be customized depending on the target brain region.

The in vivo animal experiment with transgenic mice proved the functionality of the proposed optrode system, including light delivery and electrophysiological recording. For optogenetic modulation, ChR2 was expressed mostly on excitatory neurons of the transgenic mice. The optogenetic activation increased the frequency of spikes approximately two-fold compared to the spontaneous activity frequency. Furthermore, time-locked spiking in the stimulation region was observed. This was because the target region, namely, the hippocampus, has a signal pathway from hippocampal CA3 to CA1. The hippocampus was chosen as a target brain region because it is a representative brain region that is known to be an important center for learning and memory [33,34,35,36,37,38]. In the hippocampus, there exists a high density of neuronal cell bodies and a complicated formation of the neural pathway. We placed optical fibers in hippocampal CA3 and recording electrodes in hippocampal CA1. Our results can be interpreted as showing that activated excitatory neurons in CA3 transmitted neural signals to CA1.

The whole system comprises disposable and reusable parts in consideration of several aspects of in vivo animal experiments. First, the detachable design can reduce the cost of large-scale animal experiments. In most neuroscience studies, it is required to perform experiments with at least several tens of animals to verify hypotheses. Once the optrode system is implanted into the brain, it is impossible to recycle because the system is firmly attached to the skull with dental cement. Therefore, the detachable design can substantially reduce the system’s cost. Second, the detachable system is advantageous because it minimizes the total weight of implantation while the animal is not performing the experiments. In most in vivo experimental designs, the actual experiments do not exceed a couple of hours per day. In addition, it is very crucial to reduce the weight of the system since the mice cannot freely move with a heavy and uncomfortable burden on their head. Lastly, the detachable design can protect the system from animals’ attempts to remove and eventually deteriorate the implanted system.
